# Antibody-Mediated Rejection: Pathogenesis, Prevention, Treatment, and Outcomes

**DOI:** 10.1155/2012/201754

**Published:** 2012-03-24

**Authors:** Olivia R. Blume, Sarah E. Yost, Bruce Kaplan

**Affiliations:** ^1^Abdominal Transplantation, University of Arizona Medical Center, Tucson, AZ, USA; ^1^Abdominal Transplantation, Department of Pharmacy, University of Arizona Medical Center, Tucson, AZ, USA; ^3^University of Arizona College of Medicine, 1501 North Campbell Avenue, Tucson, AZ 85724, USA

## Abstract

Antibody-mediated rejection (AMR) is a major cause of late kidney transplant failure. It is important to have an understanding of human-leukocyte antigen (HLA) typing including well-designed studies to determine anti-MHC-class-I-related chain A (MICA) and antibody rejection pathogenesis. This can allow for more specific diagnosis and treatment which may improve long-term graft function. HLA-specific antibody detection prior to transplantation allows one to help determine the risk for AMR while detection of DSA along with a biopsy confirms it. It is now appreciated that biopsy for AMR does not have to include diffuse C4d, but does require a closer look at peritubular capillary microvasculature. Although plasmapheresis (PP) is effective in removing alloantibodies (DSAs) from the circulation, rebound synthesis of alloantibodies can occur. Splenectomy is used in desensitization protocols for ABO incompatible transplants as well as being found to treat AMR refractory to conventional treatment. Also used are agents targeted for plasma cells, B cells, and the complement cascade which are bortezomib rituximab and eculizumab, respectively.

## 1. Understanding HLA

There are 20 class I genes and in transplantation HLA-A, B, and C are the classic genes referenced to when typing the recipient [1]. Class I major histocompatibility complex (MHC) molecule processing and loading of peptides occurs in all nucleated cells, where class II MHC molecule involves primarily B cells, macrophages, and dendritic cells by method of endocytosis and phagocytosis. Class II MHC molecule nomenclature is designated by class (D), family (M, O, P, Q, R), and chain (A or B). Late antibody-mediated rejection (AMR) is a major cause of late kidney transplant failure and several developments in understanding of pathogenesis allow for improvement of diagnosis, treatment, and prevention. This review will concentrate on reviewing the pathogenesis, diagnosis, and treatment of AMR.

## 2. Pathogenesis of HLA Antibodies

Not only can antibodies form against HLA molecules, but to endothelial-cell antigens and across ABO blood group [2]. Sensitization prior to transplant can occur by pregnancy or blood transfusions. Previous transplantation can also sensitize patients against HLA molecules. Blood transfusions can induce humoral immunity by formation of HLA alloantibodies and are more likely to occur in individuals who have been previously pregnant. Our understanding of how blood transfusions cause sensitization is incomplete, but it is not inevitable and can be attenuated by immunosuppression. In a study performed by Eikmans et al. it was found two weeks after blood transfusion in both nonsensitized and sensitized recipients, increased numbers of IFN-*γ*-producing cells were produced stimulating CD4+ and NK cells [3]. With de novo HLA production, antibody rejection can occur within weeks of the transplant. Endothelium of donor graft peritubular and glomerular capillaries display MHC molecules which are the target for antibody production. Once the endothelium is damaged by antibody, von Willebrand factor and P-selectin are released as an inflammatory response. Leukocytes adhere to glomeruli or to dilated peritubular capillaries via cytokines (IL-1 *α*, IL-8, and chemokine ligand 2) allowing complement activation. In antibody rejection, C4d is a useful marker of complement activation and is seen commonly in peritubular capillaries with proper immunohistochemical staining technique. Chemoattractants C3a and C5a are a part of the compliment cascade which activate C5b allowing for assembly of membrane attack complex (MAC) which is composed of C5b-C9. The MAC complex once activated will cause local necrosis allowing detachment of endothelial cells from the basement membrane, which is also a characteristic finding upon biopsy indicative of AMR. If not treated properly in a timely manner, sequelae can include thrombotic microangiopathy (TMA) causing hemorrhage with arterial wall necrosis, and ultimately graft loss. Also potentially playing a role in AMR pathogenesis are polymorphic MHC-class-I-related chain A (MICA) antigens [4]. It has been found that not all patients with AMR have anti-HLA antibodies, which suggests other possible mechanisms involved in acute or chronic graft damage. In a study conducted by Worthington et al., only five out of 14 patients with renal graft dysfunction and pathology showing C4d deposition in peritubular capillaries were found to have anti-HLA antibodies [5]. MICA antigens are structurally similar to MHC class I proteins, closely linked to HLA-B and C loci [6]. MICA antigens can be found on fibroblasts, endothelial cells, dendritic cells, and many tumors which unfortunately makes detection difficult by standard donor and recipient crossmatching. MICA eplet repertoire has become the basis for developing HLAMatchmaker program which analyzes antibody patterns with single MICA alleles on Luminex platform. It has been determined so far (Duquesnoy et al.) that there are 38 potentially immunogenic MICA eplets associated with MICA-reactive serum [6]. MICA and HLA class I chains are similar in configuration however none of the polymorphic residues are shared. HLAMatchmaker program is therefore used to determine the specificity of anti-MICA antibodies in sensitized patients since MICA is considered as a separate alloantigenic system. In a study by Zhang et al., 52 patients transplanted who were crossmatch negative were placed on triple therapy (cyclosporine/tacrolimus, mycophenolate, and prednisone). Once graft dysfunction was diagnosed with positive C4d, both anti-HLA and anti-MICA antibodies were collected from serum and reviewed via Luminex. Of the 15 patients found to have anti-MICA antibodies, 9 patients were positive for class I and/or class II anti-HLA antibodies. Nine out of 15 patients were found to have C4d deposition, and after reviewing patients for the follow-up period (1 year), it was found that eGFR decreased in anti-MICA and anti-HLA antibody positive group. This study suggested that patients with MICA antibodies have a more rapid deterioration of graft function versus patients with just anti-HLA antibodies. There are several proposed mechanisms for anti-MICA antibody activation involving complement or natural killer cells and studies are continuing to determine the pathogenesis. A more thorough understanding of HLA typing as well as continuing studies to determine anti-MICA and antibody rejection pathogenesis may allow for novel treatment strategies.

AMR occurs in up to 33% of cases undergoing ABO incompatible kidney transplantation. Among cases in which AMR is triggered in the early post-op period, prevalence of transplant glomerulopathy is 22% at one year [7]. This is opposed to the case in which Gloor found patients who received desensitization to HLA antibodies showed more glomerular changes versus ABO incompatible transplant grafts not having clinical AMR, suggesting subclinical AMR may exist [8]. The resistance of the graft to AMR in spite of the presence of antibodies against donor endothelium is called accommodation. There are several proposed mechanisms to describe this phenomenon. One proposed mechanism is a decrease in antigen-antibody interaction. This is explained either by difference in antigen expression, change in repertoire/level of antibodies in recipient, or decreased susceptibility of injury to endothelium secondary to continual stimulation of antibody and complement components. It is proposed that continued stimulation of endothelial cells by endotoxin or IL-1 over time diminishes cellular responses to restimulation [7]. Another proposed mechanism explaining accommodation could be a decrease in the recipient's type blood-type antigen on the endothelium of the graft. Koestner et al. reported histo-blood type change of a blood type B graft after ABO-mismatched heart transplantation, where the recipient's blood-type O antigen was replaced by the donor's type B antigen after AMR graft loss [9]. The mechanism of replacement of the recipient's antigens remains unclear, and several studies have been attempted to explain occurrence of blood-type chimerism. One explanation is replacement of the recipient type antigens by stem cells derived from injured vessel walls after graft rejection. It is speculated that during AMR some blood-type antigens could be shaved off from endothelial surface from immunological reaction, resulting in loss of blood-type antigens on the transplanted mismatched organ. In addition the presence of diffuse C4d without capillary damage or inflammation infers that accommodation may include resistance to the terminal complement cascade. There are some noted differences between HLA-sensitized and ABO-incompatible renal transplantation in terms of pattern of cell injury after C4d deposition. It has been found that there is more graft loss with positive C4d AMR in HLA-sensitized patients which may be due to differences in biological responses between non-self-carbohydrate antigens (blood type antigens) and non-self-peptide antigens (HLA antigens) [9].

## 3. Prevention

Crossmatching not only improves long-term graft survival by identifying the presence of antidonor antibody in the recipient, but is used as a predictor of AMR [10]. Flow crossmatch allows determination of positive T- or B-cell crossmatch for transplantation. The donor cells are incubated with the recipient serum, and antihuman globulin which is fluorescent dye tagged is placed in the flow cytometer. Positive T-cell crossmatch predicts rejection more accurately than B-cell crossmatching. HLA-specific antibody detects antibodies binding to individual HLA antigens and allowing determination of antigens to avoid in a donor, coined “unacceptable antigens” thus avoiding rejection. Of patients who are highly sensitized, 30% can develop AMR [10]. HLA-specific antibody detection prior to transplantation and surveillance after transplantation during a rejection episode allows for avoidance of rejection and determination of course of treatment for AMR, respectively [11].

## 4. Diagnosis

The 1997 Banff classification was used to classify rejection prior to the meeting in 2001, which further defined pathological classification of AMR [12]. Gorer determined the role of antibody after much debate in the early days of transplantation, and unfortunately after his death in 1961 the concept of AMR was lost. In 1991-1992, Feucht and Halloran described C4d as a marker for AMR however this was not uniformly utilized until the report from Solez et al. in 1998–2000 [13]. After international transplant meetings in 1997, AMR was an entity recognized by most of the participants and C4d staining raised hope that morphological classification of AMR can be further defined. Incidence of AMR has been reported as 0–8% in renal transplant recipients in larger centers largely due to increased recognition, detection of DSA, retransplanted patients, as well as increase in positive crossmatch and ABO incompatible transplantation for highly sensitized patients. Colvin had reviewed studies from Massachusetts General Hospital which for the first time indicated a clear correlation of peritubular capillary C4d staining pathological features with DSA in AMR. A few studies have indicated C4d staining is around 93–96% specific, but 31–95% sensitive [12]. This raises a concern that additional evidence is needed to diagnose AMR such as quantification of DSA or other morphologic feature consistent with AMR on pathology. However it should be noted that Regele and colleagues presented data from 1 year after AMR that kidney transplant patients with positive C4d had higher incidence of graft loss and elevation of serum creatinine [14]. After the Banff meeting in 2001 it was determined that AMR has 3 cardinal features upon biopsy findings.

1. Acute tubular injury; neutrophils and/or mononuclear cells in peritubular capillaries and/or glomeruli, and/or capillary thrombosis; or arteritis/fibrinoid necrosis in the intima along with intramural/transmural arterial inflammation.
2. C4d evidence for antibody action and/or immunoglobulin in peritubular capillaries, immunoglobulin and complement in arterial fibrinoid necrosis.
3. Anti-HLA antibody (DSA) circulation in serum or other antidonor endothelial antigens.

After the Banff meeting in 2009, positive C4d deposition without evidence of rejection was added to criteria suggesting antibody-mediated changes. Several studies had reported this finding including Mark Haas (Los Angeles) who had seen C4d deposition in biopsies without evidence of rejection [15]. In protocol biopsies from ABO incompatible grafts, 21/37 had C4d deposition without evidence of AMR lesions or T-cell-mediated rejection which can suggest accommodation. Another addition to Banff criteria indicating antibody-mediated changes was determined to be positive C4d with presence of circulating antidonor antibodies (no signs of acute or chronic T-cell-mediated rejection or AMR/no ATN-like minimal inflammation).

In normal kidneys, specifically in the glomerular mesangium as well as arterioles at the vascular pole, turnover of immune complexes occurs (C4d) [16]. Transplanted kidneys in addition show deposition of C4d in peritubular capillaries and can follow a dynamic course. C4d can be in a transitional state and show staining of only a few capillaries indicating either an ongoing humoral attack or resolving previous attack. There is sometimes diffuse staining in every capillary or focal staining in only a few capillaries, but C4d is always attached to endothelial cells and basement membranes.

There has also been evidence for C4d negative AMR in a couple studies, which in the past few years has brought to the forefront the limitations of C4d as a marker for AMR [17]. In one such study by Sis et al., overexpression of 12-individual endothelial-associated transcripts genes (ENDATs) upon transplant biopsies with graft dysfunction was correlated with an increased risk of graft loss [18]. High ENDAT expression and endothelial activation with DSA was found to be strongly associated with the presence of transplant glomerulopathy. Interestingly, fewer than 50% of the biopsies performed (*n* = 173) with DSA and ENDATs had diffuse C4d staining in peritubular capillaries. Investigators also have found that C4d negative form of AMR is less severe than C4d positive AMR but is associated with chronic changes within the graft, such as transplant glomerulopathy. A possible complement-independent mechanism of microcirculation injury in AMR is plausible. It has been postulated that DSA binding to endothelial cells triggers natural-killer cells to release interferon-*γ* resulting in granule-associated toxicity.

### 4.1. Monitoring of DSA

DSA has been found to play a role in diagnosis of AMR and can be an independent predictor of allograft loss [19]. It was found when patients had DSA at time of clinical rejection diagnosed by Luminex, 50% reduction in DSA within 14 days of diagnosis had higher allograft survival. In a study performed by Everly et al., acute rejection was defined as an increase in serum creatinine by 20% above the baseline upon rejection; DSA was identified by antigen bead panels by Luminex assay [19]. 650 patients were analyzed for acute rejection, 94 of which were identified with biopsy-proven acute rejection by Banff criteria. Fifty-two of these patients were found to have DSA present. Analysis of predictors of allograft loss revealed DSA to be an independent predictor of allograft loss, with a sixfold increase in allograft loss rates. However, it is noted in this study that DSA was present in the absence of C4d staining on biopsy. Further studies need to be conducted to determine if complete elimination of DSA is necessary. However, from this study it should be deduced that DSA reduction should receive consideration as a potential therapeutic goal for rejection therapy. Suppression of DSA with antihumoral therapies may provide a means for improving long-term renal allograft survival. Bortezomib, a new anti-B-cell agent that targets antibody producing cells (plasma cells) has shown promise as an effective means for reducing DSA as well. It has also been postulated that surveillance of DSA can be a means of preventing rejection, especially for highly sensitized patients [20]. It has been discussed that high-risk patients (presensitized and retransplants) may benefit from more DSA surveillance in the first 3 months after transplant to detect an early immune response; however this is not rigorously proven to improve outcomes.

## 5. Treatment

### 5.1. Plasmapheresis, Immunoadsorption, and IVIG

Plasmapheresis (PP) removes alloantibodies from the circulation and is the fastest and most effective method for elimination of DSA [11]. Usually 1.0–1.5 full-volume exchanges are performed using albumin solution daily or alternate days, continued until serum creatinine decreases 30% from the baseline value. Although PP is effective in removing alloantibodies (DSA) from the circulation, rebound synthesis of de novo alloantibodies can occur. IVIG is used in combination with PP to neutralize antibodies and potentially block complement activity as well as agents used for suppression of antibody (cacineurin inhibitors, mycophenolate, or rituximab).

Immunoadsorption (IA) is a substance derived from the Cowan strain of *Staphylococcus aureus*. The protein A coating the column beads has a strong affinity for the Fc portion of immunoglobulin and immune complexes [21]. For the past decade, protein A IA was found to remove harmful antibodies and immune complexes efficiently and has been used as treatment of AMR of highly sensitized patients. It was found in a study by Qing et al. that classes I and II panel-reactive antibody (PRA) as well as immunoglobulin and complement (C3 and C4) were reduced significantly. However, it was found that within 6 to 8 hours rebound of antibodies was seen. Previous studies have indicated though that long-lasting favorable decrease in antibodies persisted. It has been postulated that IA may cause a conformation change of the structure of the antibodies and immune complexes making them more immunogenic, and immunostimulating to regulatory anti-idiotype antibodies. These antibodies play a role in the control of autoimmune mechanisms and reverse the continuing cycle of abnormal immune balance. The long lasting response of IA therapy may be explained by phagocytosis of the reticuloendothelial system and activation of complement.

IVIG is prepared by human plasma from approximately 50,000–100,000 of healthy donors, composed of 90% intact IgG, a few dimers, Fabs (fragment antigen-binding) and traces of IgM and IgA [11]. there are many postulated mechanisms of action of IVIG. The Fc region in IgG allows it to interact and signal through Fc-gamma receptors on phagocytes, B cells, and other cells as well as with Fc-binding plasma proteins, such as components of the complement system [20]. Immune globulin also prevents damage mediated by immune complexes containing C3b into an inactive form iC3b as well as neutralization of some cytokines. IVIG can neutralize autoantibodies and down regulate synthesis of antibodies by B cells expressing the relevant idiotype. There are some studies with usage of IVIG alone or in combination with PP, and when IVIG is used alone a high dose is given (1-2 g/kg). It was found IVIG was effective in reversing rejection within 2–5 days of infusion with no recurrence in kidney transplant recipients [11]. The usual recommended dose is 100 mg/kg of IVIG after each PP session and 300–400 mg/kg for 1-2 days after the last PP with a cumulative dose of 1000 mg/kg. Various dosing schedules are used, and the optimal dose has not been established. A benefit of IVIG is its ability to replenish gammaglobulin lost during PP, decreasing infection risk. Adverse effects of IVIG which are common are headache, fever, chills, myalgias, and hypotension/hypertension which can be reduced by slowing the infusion rate. Serious adverse effects are rare and can include aseptic meningitis, acute renal failure, thrombotic events, and anaphylaxis.

### 5.2. Antilymphocyte Therapy

Jin et al. had used Thymoglobulin (ATG) 0.75 mg/kg/day for 5–10 days in combination with plasma exchange in 7 patients with AMR and showed 85% reversal in AMR [21]. ATG is a polyclonal preparation generated from the immunization of rabbits with human thymus. ATG eliminates CD4+ T-cell and B-cell interaction causing B-cell toxicity/apoptosis and modulation of alloantibody production. If both cellular and humoral features are seen on biopsy, ATG is often used along with steroids for treatment of AMR. ATG can be given in 3-4 doses to a cumulative dose of 6 mg/kg, and it is noted that leukopenia is a side effect which should be monitored to guide specific treatment for each patient.

### 5.3. Splenectomy

The spleen is the largest lymphoid organ in the body and has been found to play a role in alloantibody generation. Splenectomy is used in desensitization protocols for ABO incompatible transplants as well as positive crossmatches, however it has been found to surprisingly treat AMR refractory to treatment [22]. There are a few case studies, one in particular where abundant clusters of CD138+ plasma cells were found upon review of pathology of the spleen [22]. Usually in traumatic splenectomy controls, CD138+ cells are found in 1% of the spleen. This patient had refractory AMR without improvement in her serum creatinine. She had been treated with PP/IVIG and then underwent emergent splenectomy. Immediately afterward, there was improvement in her renal function and rapid drop in DR51 antibodies [22]. The mechanism of action is not entirely clear as to why splenectomy treats AMR, but several case reports have indicated that it could be a potential treatment for refractory AMR to PP/IVIG. It is not certain whether the CD138+ cells found in the spleen had produced anti-DR51 antibodies in this particular case, but provides a novel insight for further studies in the role of the spleen with AMR. Of course, splenectomy has risks associated with it which include increased risk of infections and the risks associated with surgery. Therefore these risks must be discussed with the patient before proceeding, as this is not a conventional treatment for AMR. There is lifelong risk with encapsulated microorganisms; therefore patients must receive *Haemophilus *influenza vaccine, pneumococcal, and meningococcal vaccines.

### 5.4. Eculizumab

Most episodes of AMR are associated by evidence of early complement activation by the presence of C4d+ staining of the peritubular capillaries [23]. The exact role of complement in the pathogenesis of AMR is still being investigated. Eculizumab is a humanized monoclonal antibody with high affinity for C5 and blocks the activation of terminal complement and is FDA approved for the treatment of paroxysmal nocturnal hemoglobinuria. Serious adverse effects of this medication are an increased risk of infections, particularly to encapsulated bacteria. It is recommended to immunize patients at least two weeks before the administration of eculizumab to meningococcus and pneumococcus.

The transplant community is looking towards the use of this medication because of its highly selective mechanism of action [24]. There are several case reports using eculizumab for desensitization before transplant and to treat AMR after transplant. Stegall and colleges reported using eculizumab in sensitized renal transplant recipients with high levels of DSA to determine if the incidence of AMR in the first 3 months after transplant was decreased compared to historical controls. These patients were immunized one month prior to transplant, received plasmapheresis treatments before and after transplant based on the crossmatch channel shifts, and received the same induction and maintenance immunosuppression. The eculizumab dosing regimen included 600 mg on postoperative day 1, and 600 mg weekly thereafter for 4 weeks. At week 4, assessment of DSA levels was performed to determine if patients would continue treatment based on DSA levels. The study found the incidence of AMR was statistically significanty lower in the eculizumab group compared to the control group and reduced the need for splenectomy, reducing transplant glomerulopathy [25]. Considering the cost of this medication, this dosing regimen and prophylactic use might be unrealistic for smaller transplant centers. A few case reports used eculizumab in treating renal thrombotic microangiopathy (TMA) and reversal of complement activation after ABO-incompatible transplantation [26–28]. The use for TMA involved a patient who underwent a living related kidney transplant with a history of lupus developed severe TMA, glomerular scarring, and diffuse tubulointerstitial fibrosis with positive aPL antibodies. The patient received dialysis and plasmapheresis sessions with no improvement in kidney function. The patient received weekly infusions of eculizumab without complication and renal function improved with subsequent biopsy revealed complete resolution of TMA [26].

### 5.5. Bortezomib

The transplant community has recognized the potential use of an agent that can directly target plasma cells. Traditional treatments have been successful in removing antibodies, inhibiting antibody activity or lowering production but no treatments have been effective in targeting mature antibody production in plasma cells. AMR involves the production of high levels of DSA by plasma cells either newly made from memory or naïve B cells or from those that existed prior to transplant. One mechanism to control AMR is to target DSA production by plasma cells. Bortezomib is a proteasome inhibitor which is FDA approved for the treatment of multiple myeloma. This medication has been shown to cause apoptosis of normal plasma cells which in turn decreases alloantibody production in sensitized patients [28].

Several case reports and series have been published regarding the use of bortezomib. Currently available studies of bortezomib for treatment of AMR are summarized in Table 1. The University of Cincinnati reported the first study on the use of bortezomib in AMR. Everly and colleagues treated six kidney transplant recipients with refractory AMR and concomitant ACR with bortezomib and found fast rejection reversal, reductions in DSA levels, improved renal allograft function, and suppression of recurrent rejection [29]. The same group reported two patients with AMR who received bortezomib and these patients experienced prompt AMR reversal and DSA elimination in 14 days [30]. The use of bortezomib has also been published in five patients with a combination of ACR and AMR. These patients were given four doses of bortezomib and reversal of both ACR and AMR occurred with reduction in DSA [30]. Other studies have shown positive and mixed results regarding the use of this medication in AMR and DSA levels [31, 32].

### 5.6. Rituximab

Rituximab is a chimeric anti-CD 20 (anti-B cell) monoclonal antibody that is currently FDA approved for the treatment of lymphoma. The CD 20 antigen is expressed early in B-cell cycle but is absent on mature plasma cells. The variable region of rituximab binds to CD 20 through three different mechanisms including antibody-dependent cell cytotoxicity, complement-dependent cell killing, and induction of apoptotic cell death. These mechanisms mark cells for destruction and leads to sustained depletion of circulating B cells [32, 33]. Genberg and colleagues reviewed the pharmacodynamics after a single dose of rituximab given to renal transplant patients and showed B-cell elimination was rapid and occurred in the peripheral blood over 1-2 days. The effect on the B-cell population was prolonged and did not reemerge for 1 year and remained suppressed for 2 years [34].

The first report of using rituximab in AMR evaluated 27 patients with refractory rejection and received a single dose of rituximab. Three grafts were lost and the remaining grafts had good function at the time of discharge [35]. Kaposztas and colleagues published a retrospective study involving 54 patients with AMR and were split into two groups. Group A (*n* = 26) received plasmapheresis and rituximab and Group B (*n* = 28) received plasmapheresis alone. Patients with low serum IgG levels also received immunoglobulin. The two year graft survival was significantly better in the rituximab group [36]. Since these two large case reports, there have been many case series, and so forth published on using rituximab with any of the studies for desensitization and for AMR [37–40]. With many of studies the patient population is small, with incomplete criteria for AMR diagnosis, and other treatments used in conjunction with rituximab including IVIG, plasmapheresis, and steroids. However it should be noted in other studies particularly by Pescovitz, a small number of patients failed to show a decrease in PRA with rituximab alone [41].

## Figures and Tables

**Table 1:** Published studies of the use of bortezomib in AMR [11].

Study	*N*	Patients	Treatment	Outcome
Wade et al. 2009 [40]	5	Renal transplant with mixed AMR and ACR	Bortezomib 1.3 mg/m^2^/dose × 4	(i) Prompt AMR and ACR reversal (ii) Significant decrease in DSA levels

Tanriover et al. 2008 [38]	6	Kidney/kidney pancreas transplant with mixed AMR and ACR	Same as above	(i) Prompt rejection reversal marked and prolonged reductions in DSA levels (ii) >50% decrease in DSA levels within 14 days and suppression for up to 5 months

Celik et al. 2009 [37]	2	Positive crossmatch renal transplant recipient with AMR	Bortezomib 1.3 mg/m^2^/dose on days 1, 4, 8, 11 Daily plasmapheresis and IVIG	(i) Decrease HLA allospecificities (ii) Decrease in number of plasma cells in bone marrow

Faguer et al. 2010 [39]	4	Renal transplant recipients with AMR and persistently elevated DSA	Bortezomib 1.3 mg/m^2^/dose × 4	No significant decrease in DSA within 150 days post treatment

Wade et al. 2009 [40]	11	Living donor renal transplant patients with anti-HLA alloantibodies	Bortezomib 1.3 mg/m^2^/dose with methylprednisolone 250 mg on days 1, 4, 8, 11 2–4 sessions of plasmapheresis 1 dose of rituximab (6 patients)	(i) Reduced DSA and non-DSA levels (ii) Stable graft function 4 months post treatment

Faguer et al. 2010 [40]	2	Living donor renal transplant	Bortezomib 1.3 mg/m^2^/dose × 4 Ongoing plasmapheresis, rituximab, intravenous steroids	(i) Immediate significant reduction in DSA (ii) Stable graft function at 6 months post treatment
